# Frailty in Cardiorenal Care: A Geriatric Perspective on the 2026 European Society of Cardiology Guidelines

**DOI:** 10.7759/cureus.115928

**Published:** 2026-09-08

**Authors:** Aleksandr Martynenko

**Affiliations:** 1 Department of Internal Medicine, LLC "Multifunctional Medical Center" M-Clinic, Tashkent, UZB

**Keywords:** 2026 esc guidelines, biological aging, cardiorenal risk, cardiovascular disease, chronic kidney disease, frailty, geriatric cardiology, healthy longevity, older adults, polypharmacy

## Abstract

The 2026 European Society of Cardiology Guidelines for the Management of Cardiovascular Disease and Chronic Kidney Disease provide an integrated framework for cardiorenal risk detection, treatment modification, and health service planning. However, chronological age, kidney function, albuminuria, and conventional cardiovascular risk measures cannot fully capture the clinical heterogeneity of older adults. Frailty, sarcopenia, cognitive impairment, multimorbidity, polypharmacy, and limited life expectancy may substantially alter both the expected benefits and burdens of treatment. Conversely, advanced age alone should not justify withholding evidence-based cardiorenal therapy from functionally preserved older adults. This editorial proposes STAMP-F as a conceptual and currently unvalidated extension of the guideline framework. The additional component represents Frailty, Function, and Future Healthspan and is intended to support individualized decisions rather than serve as an official recommendation, diagnostic criterion, or prognostic instrument. Brief measures such as the Clinical Frailty Scale and gait speed may provide pragmatic entry points for this assessment. Incorporating these domains may help align organ-protective treatment with outcomes that matter to older adults, including mobility, cognition, independence, quality of life, and healthspan. Prospective studies are needed to determine whether this approach improves clinical decision-making and patient-centered outcomes.

## Editorial

The new cardiorenal framework

The 2026 European Society of Cardiology Guidelines for the Management of Cardiovascular Disease and Chronic Kidney Disease represent an important change in clinical practice. For the first time, the European Society of Cardiology has issued dedicated guidance that treats chronic kidney disease (CKD) not merely as a comorbidity but as an integral component of cardiovascular medicine. The central STAMP on CKD framework stands for Screen, Triage, and Address CKD risk, Modify cardiovascular disease management, and Plan health services. It promotes earlier detection of CKD, risk stratification, appropriate use of cardiorenal therapies, modification of cardiovascular management, and coordinated service planning [1].

This framework is particularly relevant to aging populations. Cardiovascular disease and CKD become increasingly prevalent with advancing age and frequently coexist with hypertension, diabetes, obesity, atrial fibrillation, anemia, and multimorbidity. However, older adults are not a biologically homogeneous population. Two patients aged 80 years with similar estimated glomerular filtration rates and albuminuria may have profoundly different physiological reserves, life expectancies, treatment priorities, and capacities to tolerate interventions.

One patient may remain physically active, cognitively intact, and independent. Another may have sarcopenia, recurrent falls, orthostatic hypotension, cognitive impairment, and dependence in daily activities. Similar cardiorenal risk does not necessarily require an identical clinical decision. Kidney function, albuminuria, and conventional cardiovascular risk estimates remain essential, but they do not fully describe the vulnerability or resilience of an older person.

Chronological age is not a treatment strategy

Chronological age is used inconsistently in clinical practice. In some patients, it leads to therapeutic inertia and the inappropriate withholding of interventions that may reduce cardiovascular and kidney events. In others, guideline targets are pursued aggressively despite severe frailty, symptomatic hypotension, limited life expectancy, or an excessive treatment burden. Both approaches risk substituting chronological age for biological age.

Biological aging reflects the progressive loss of physiological resilience associated with interconnected processes such as chronic inflammation, cellular senescence, mitochondrial dysfunction, endothelial injury, and impaired repair mechanisms. These processes contribute simultaneously to cardiovascular disease, declining kidney function, sarcopenia, cognitive impairment, and frailty [2]. Cardiovascular disease and CKD in an older person should therefore be considered not only as disorders of two organs but also as interacting manifestations and potential accelerators of systemic biological aging.

CKD may increase functional vulnerability through inflammation, oxidative stress, anemia, metabolic acidosis, vascular calcification, nutritional disturbances, and the accumulation of uremic toxins. Cardiovascular disease can further impair exercise tolerance, mobility, and physiological reserve. This creates a bidirectional relationship in which cardiorenal disease contributes to functional decline, while frailty reduces the ability to tolerate acute illness, hospitalization, procedures, and complex pharmacotherapy.

This relationship has independent prognostic importance. A large systematic review and meta-analysis found frailty in approximately one-third of patients with CKD, while prefrailty affected an additional substantial proportion. Frailty was associated with an almost twofold increase in mortality [3]. Another systematic review and meta-analysis linked frailty in CKD with mortality, hospitalization, and falls [4]. These outcomes are not peripheral to cardiorenal medicine. They often determine whether an older person remains independent.

From STAMP to STAMP-F

The 2026 guidelines acknowledge frailty, multimorbidity, polypharmacy, treatment tolerability, psychological burden, and patient preferences in several clinical contexts. They also recognize that older adults and functionally vulnerable patients have often been underrepresented in randomized trials [1]. However, these considerations are distributed across different sections and are not incorporated as a systematic geriatric step within the central STAMP pathway.

We therefore propose STAMP-F as a conceptual extension of the guideline framework. The additional component represents Frailty, Function, and Future Healthspan. STAMP-F is not an official European Society of Cardiology recommendation, a diagnostic criterion, or a validated prognostic instrument. It is a pragmatic and testable framework intended to support the individualized implementation of STAMP in older adults.

Frailty should not be determined by an unstructured impression that a patient "looks frail". It should involve a brief standardized assessment of physiological reserve using a validated instrument appropriate to the clinical setting. Pragmatic options include the Clinical Frailty Scale and a brief performance-based measure such as gait speed; abnormal findings can prompt more detailed assessment. Such an assessment is particularly relevant when an older patient has unintentional weight loss, reduced mobility, exhaustion, recurrent falls, repeated hospitalizations, or difficulty managing medications.

Function means that mobility, cognition, and independence should be considered clinically meaningful outcomes rather than background characteristics. A pragmatic evaluation may examine basic and instrumental activities of daily living, falls, nutritional status, cognition, mobility, and the ability to follow a treatment regimen. The purpose is not to create an additional administrative burden but to identify vulnerabilities that may alter the expected benefits, risks, or feasibility of treatment.

Future Healthspan refers to the anticipated period of life without severe disability or dependence. It requires clinicians to consider time to benefit, life expectancy, competing risks, treatment burden, and the patient's priorities. After cardiorenal risk has been established, the clinician should additionally ask whether frailty is present, how the proposed intervention may affect function, and whether the patient is likely to experience its benefits within a clinically meaningful time frame.

This approach may help distinguish three broad clinical situations without creating rigid treatment categories. A functionally preserved older adult should receive evidence-based cardiorenal prevention regardless of chronological age. A patient with potentially modifiable functional vulnerability may require cardiorenal treatment combined with nutritional support, physical activity, correction of anemia, management of depression, and medication review. In a patient with advanced frailty and limited life expectancy, priorities may shift toward safety, symptom control, treatment simplification, and quality of life. These scenarios are intended to guide clinical reasoning rather than define fixed thresholds, and the clinical utility of STAMP-F requires prospective evaluation.

Avoiding both undertreatment and overtreatment

A geriatric assessment must not become a rationale for withholding evidence-based therapy from older adults. The 2026 guidelines report that the benefits of newer cardiorenal therapies appear generally consistent across age groups and among patients with frailty, multimorbidity, and polypharmacy [1]. Functionally preserved older adults should not be excluded from organ-protective treatment solely because of chronological age.

At the same time, relative risk reductions should not be interpreted without considering absolute benefit, competing mortality, treatment tolerability, affordability, and patient preferences. A medication may reduce the risk of a future cardiovascular or kidney event while contributing to symptomatic hypotension, dehydration, unwanted weight loss, or a regimen that is too complex for a cognitively vulnerable patient. The appropriate response is usually not automatic treatment withdrawal but careful initiation, monitoring, medication simplification, and reassessment. In frail adults, this may also require individualized blood pressure and glycemic targets and, where clinically appropriate, closer monitoring of volume status, kidney function, and serum potassium after treatment initiation or dose adjustment.

Polypharmacy deserves particular attention. Cardiorenal management may include renin-angiotensin system inhibitors, sodium-glucose cotransporter 2 inhibitors, mineralocorticoid receptor antagonists, diuretics, lipid-lowering drugs, antithrombotic therapy, and glucose-lowering agents. Each treatment may be individually justified, while the combined regimen becomes unsafe or unmanageable. The introduction of high-value therapies should therefore be accompanied by a review of the entire medication list and the deprescribing of treatments that provide little benefit or create avoidable harm.

Frailty itself should be treated as a dynamic state rather than an irreversible endpoint. Exercise, nutritional support, correction of anemia, medication optimization, and prevention of hospital-associated deconditioning may reduce functional vulnerability in some patients. This perspective is consistent with the 2024 Kidney Disease: Improving Global Outcomes guideline, which emphasizes personalized care across the life course and recognizes that diagnostic approaches, treatment goals, and decision-making may differ at advanced ages [5].

When vulnerability spans multiple domains, a more comprehensive geriatric assessment may be appropriate. The Geriatrics 5Ms, Mind, Mobility, Medications, Multicomplexity, and Matters Most, provide a concise structure for organizing such assessment [6]. In CKD care, the POLDER implementation study showed that a nephrology-tailored geriatric assessment could be integrated into routine care and was considered relevant by healthcare professionals; collaboration with geriatric departments was also perceived as valuable [7].

From organ protection to healthy longevity

Cardiorenal trials and quality indicators predominantly assess mortality, cardiovascular events, hospitalization, decline in estimated glomerular filtration rate, and kidney failure. These outcomes are essential, but they do not fully capture what matters to many older adults. Future studies evaluating the implementation of the 2026 guidelines should also measure mobility, falls, cognition, activities of daily living, quality of life, treatment burden, and loss of independence.

The STAMP-F concept creates a testable research agenda. Prospective studies should determine whether adding standardized assessments of frailty and function to cardiorenal risk evaluation changes therapeutic decisions, improves treatment tolerability, prevents functional decline, or better aligns care with patient priorities. Research should also establish which brief instruments are feasible across primary care, cardiology, nephrology, and resource-limited healthcare systems.

The 2026 European Society of Cardiology Guidelines provide a landmark foundation for integrated cardiovascular and kidney care. Their implementation in aging populations should account for biological rather than chronological age and should avoid both age-based undertreatment and treatment that imposes disproportionate burdens on vulnerable patients.

STAMP-F extends the organ-protection focus of STAMP by adding Frailty, Function, and Future Healthspan. It should be regarded as a conceptual and testable extension rather than an official recommendation or a completed prognostic tool. Its value will depend on prospective validation and its ability to improve decisions that preserve mobility, cognition, independence, and healthy longevity.
